# Rg6, a rare ginsenoside, inhibits systemic inflammation through the induction of interleukin-10 and microRNA-146a

**DOI:** 10.1038/s41598-019-40690-8

**Published:** 2019-03-13

**Authors:** Seungwha Paik, Jin Ho Choe, Ga-Eun Choi, Ji-Eun Kim, Jin-Man Kim, Gyu Yong Song, Eun-Kyeong Jo

**Affiliations:** 1 Department of Microbiology, Chungnam National University School of Medicine, Daejeon, 35015 Republic of Korea; 2 Department of Medical Science, Chungnam National University School of Medicine, Daejeon, 35015 Republic of Korea; 3 Infection Control Convergence Research Center, Chungnam National University, Daejeon, 35015 Republic of Korea; 4 College of Pharmacy, Chungnam National University, Daejeon, 34134 Republic of Korea; 5 Department of Pathology, Chungnam National University School of Medicine, Daejeon, 35015 Republic of Korea

## Abstract

The immunobiological functions of Rg6, a rare ginsenoside from ginseng, have been largely unreported. In this paper, we demonstrate that Rg6 has a significant immunosuppressive function on Toll-like receptor (TLR) 4-induced systemic inflammatory responses. Rg6 was found to negatively regulate pro-inflammatory responses and severity *in vivo*, and thus induced recovery in mice with lipopolysaccharide (LPS)-induced septic shock and cecal ligation and puncture (CLP)-induced sepsis. Rg6 treatment also facilitated recovery in mice with LPS-induced lung damage via reduced neutrophil infiltration and tumor necrosis factor-α expression in lung tissues. Rg6 injection also downregulated pro-inflammatory cytokines and increased the levels of interleukin (IL)-10 in the serum of septic mice. Mechanistically, Rg6 did not induce TLR negative regulators, such as A20 and IRAK-M, in bone marrow-derived macrophages (BMDMs). Instead, addition of Rg6 to LPS-activated BMDMs augmented IL-10 expression, whereas it inhibited inflammatory signaling, such as by nuclear factor κB activation and mitogen-activated protein kinases. Furthermore, Rg6 significantly induced miR-146a, an operator miRNA for anti-inflammation, in BMDMs. Collectively, these data indicate that Rg6 inhibits inflammatory responses through the induction of IL-10 and miR-146a.

## Introduction

Sepsis is the cause of one-third of the deaths of hospital patients^1–3^. It is a life-threatening condition caused by infection, accompanied by a strong inflammatory response, which is called a cytokine storm, involving tissue and organ damage during the acute phase of sepsis. The incidence of sepsis appears to be increasing, especially in older patients due to the prevalence of weakened immune systems and the use of immunosuppressive agents^4,5^. However, the main management protocol for sepsis relies on symptomatic and supportive treatment. Thus, the development of new therapeutic strategies for the treatment of sepsis through reduction of the cytokine storm and reversal of organ injuries without any prominent side effects is greatly recommended^6,7^.

One host defense that is activated to prevent excessive inflammation is the production of anti-inflammatory cytokines, such as interleukin-10 (IL-10)^6,8^. By increasing IL-10 levels, the host can resolve the inflammatory state and control body homeostasis. Many reports have demonstrated that IL-10 is a major host defense mediator against sepsis-induced impairment^9,10^. The induction of IL-10 may not only reduce the levels of pro-inflammatory cytokines, but has also been demonstrated to control innate immunity by clearing bacteria from pathogen-infected lungs and improving the survival of the host^9^. Moreover, one of the primary causes of sepsis is lipopolysaccharide (LPS), a main component of the Gram-negative bacterial cell wall^11,12^. It has been demonstrated that LPS activates Toll-like receptor (TLR) 4, resulting in the phosphorylation of nuclear factor κB (NF-κB)^12,13^. The activation of NF-κB regulates the levels of various pro-inflammatory cytokines, including tumor necrosis factor alpha (TNF-α), interleukin-6 (IL-6), and interleukin-1β (IL-1β). Thus, obstruction of the NF-κB signaling pathway is another treatment option to downregulate excessive inflammatory responses in patients with LPS-induced septic shock.

Recently, microRNA (miRNA) miR-146a was found to be an immune regulator^14^. By targeting TRAF6 and IRAK1, which are downstream molecules in the TLR signaling pathway, miR-146a controls inflammatory cytokine signaling through a negative feedback regulation loop. This miRNA has been established as a potent negative regulator of inflammation^15–17^. Research on the characteristics and functions of miR-146a has advanced to other fields, including autoimmunity and cancer^18^.

Ginseng, which is the root of *Panax ginseng* C. A. Meyer, has long been used as a health supplement and traditional medicine in East Asian countries. Ginsenosides, also known as ginseng saponins, are one of the major components of ginseng, and several ginsenosides and their metabolites reportedly exhibit significant biological activity, including anti-inflammatory reponses^19–21^. Over 50 kinds of ginsenosides have been found. However, the characteristics of several rare ginsenosides have not yet been elucidated.

Our study demonstrates the anti-inflammatory functions of the ginsenoside Rg6, which is a very rare ginsenoside, in an LPS-induced sepsis model. To our knowledge, this is the first report of the functional effects of Rg6 against systemic inflammation. Treatment with Rg6 may effectively increase the survival of mice with LPS- and cecal ligation and puncture (CLP)-induced sepsis. Also, LPS-induced lung damage in mice was recovered by treatment with Rg6, resulting in the downregulation of pro-inflammatory cytokines and upregulation of IL-10 expression in serum. In addition, we demonstrated that treatment with ginsenoside Rg6 alone was able to significantly increase the expression of miR-146a in murine macrophages.

## Results

### Manufactured rare ginsenoside Rg6 exhibits superior anti-inflammatory effect over commercial ginsenosides

**Figure 1:**
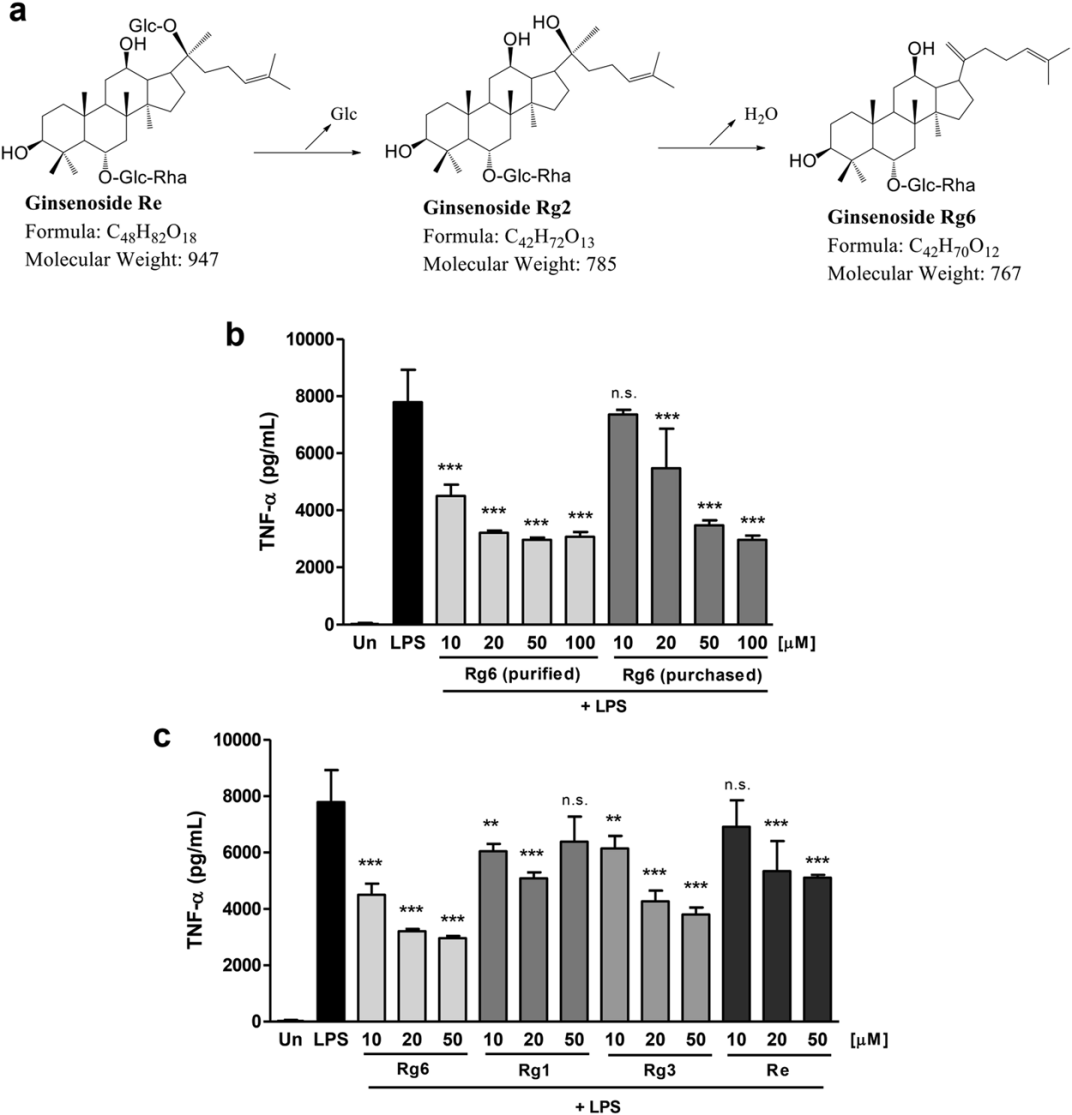
Manufactured rare ginsenoside Rg6 exhibits superior anti-inflammatory effect over previously reported ginsenosides. (**a**) New method for producing ginsenoside Rg6. The reaction scheme shows the transform process of ginsenoside Re into PPT type rare ginsenoside Rg6. (**b**) BMDMS were pre-treated with either newly produced or purchased Rg6 (10, 20, 50 and 100 μM) for 1 h, followed by LPS treatment (100 ng/mL). After 18 h, the supernatants were harvested and diluted appropriately to measure TNF-α cytokine level. (**c**) Purchased Rg1, Rg3, and Re (10, 20, and 50 μM) were treated to BMDMs to compare its activity with purified Rg6 (10, 20, and 50 μM). After 1 h, LPS (100 ng/mL) was treated to cells, and the supernatants were harvested at 18 h post-LPS treatment. The protein expression levels were measured using mouse TNF-α ELISA kit. The results are the means ± SD of at least four independent data points. Significant differences from the LPS-treated group are indicated by asterisks (**P < 0.01 and ***P < 0.001). n.s., non-specific.

The rare ginsenoside Rg6 is a specific protopanaxatriol (PPT)-type ginsenoside that exists only in black ginseng (BG)^22^. Since the amount of the rare ginsenoside Rg6 in BG is very low, we developed a new manufacturing method to produce Rg6 from the ginsenoside Re^23^, which is one of the major ginsenoside component in the fresh or white ginseng (Fig. 1a). After analyzing the purity of newly produced ginsenoside Rg6 by HPLC system (Supplementary Fig. S1), BMDMs were treated with either purified or purchased Rg6 to evaluate their anti-inflammatory activity against LPS stimulation. LPS-induced TNF-α levels significantly decreased under Rg6-treated conditions in a dose-dependent manner compared to that in the LPS only-treated group (Fig. 1b). There was no significant difference in the activity of Rg6 in the inhibition of TNF-α production in BMDMs between the commercial product and ours at high doses; rather, our purified Rg6 appeared to be more effective at low doses (e.g., 10 and 20 μM) than the commercial one. To compare the anti-inflammatory effect of Rg6 with other previously reported ginsenosides, such as Rg1^24^, Rg3^25^, and Re^23^, we examined the effect of ginsenosides on TNF-α production in LPS-activated BMDMs (Fig. 1c). The activity of Rg6 in the inhibition of TNF-α production was the greatest at all experimental doses tested, as compared to other ginsenosides. Thus, these results suggest that the anti-inflammatory activity of Rg6 prepared in the current study was comparable or superior to that of other ginsenosides, including Rg1, Rg3, and Re.

### Rg6 enhances the survival of mice in LPS- and CLP-induced sepsis

**Figure 2:**
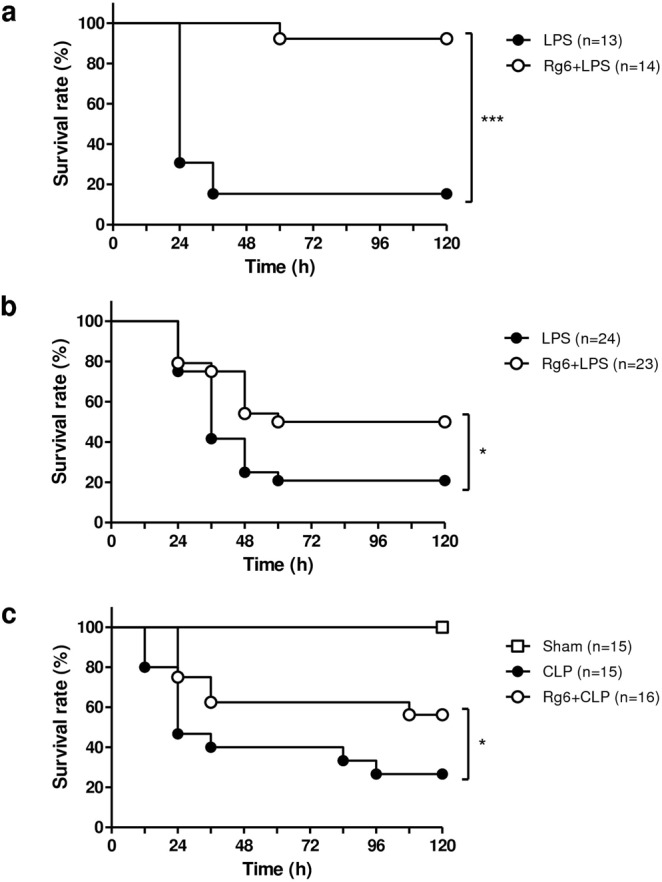
Ginsenoside Rg6 improves survival of septic mice. (**a**) Mice were IP injected with either Rg6 (20 mg/kg) or vehicle (PBS). After 2 h, a lethal dose of LPS (30 mg/kg; IP) was administered to both groups. (**b**) After administrating LPS (30 mg/kg; IP) to mice, Rg6 (20 mg/kg) or vehicle (PBS) were IP injected to each group for twice at 1 and 2 h post-LPS injection. (**c**) Mice were IP injected with either Rg6 (20 mg/kg) or vehicle (PBS). After 2 h, the mice were anesthetized and CLP procedure was performed. Sham mice were operated in the same way as CLP mice except the ligation and puncture process. The viability of these mice was assessed every 12 h until 120 h post-LPS administration or CLP procedure. Significant differences between two survival curves are indicated by asterisks (*P < 0.05 and ***P < 0.001).

To evaluate the function of Rg6 in the treatment of sepsis, we used two different murine sepsis models. In the LPS-induced sepsis model, we pre-injected mice with Rg6 (20 mg/kg) 2 h prior to intraperitoneal (IP) injection of LPS (30 mg/kg). Within 36 h, over 80% of the control mice died, whereas the overall mortality of Rg6-injected mice was less than 10% (Fig. 2a). We also post-injected Rg6 to evaluate the therapeutic effects in an LPS-induced sepsis model. After LPS (30 mg/kg) administration, Rg6 (20 mg/kg) was IP injected twice and the survival rate of mice was analyzed. Although the result was not as dramatic as in pre-treatment system (Fig. 2a), there was a significant difference in survival between the LPS control and Rg6 post-treated groups (Fig. 2b). These data suggest that Rg6 has both preventative and therapeutic potential over endotoxemia. Next, a CLP procedure was performed as described in the Materials and Methods section. IP injection of Rg6 was performed 2 h prior to the CLP procedure, as in Fig. 2a. There was also a significant difference between the control and Rg6-injected groups, with a survival rate increase of over 20% (Fig. 2c). The survival rate of the sham control was 100% until 120 h after the procedure, which demonstrated that exposing the cecum outside of peritoneal cavity had no effect on the mortality of the mice during the time they were monitored. Collectively, Rg6 enhanced the survival of LPS- and CLP-induced septic mice.

### Rg6 reduces lung damage and neutrophil infiltration in septic mice

**Figure 3:**
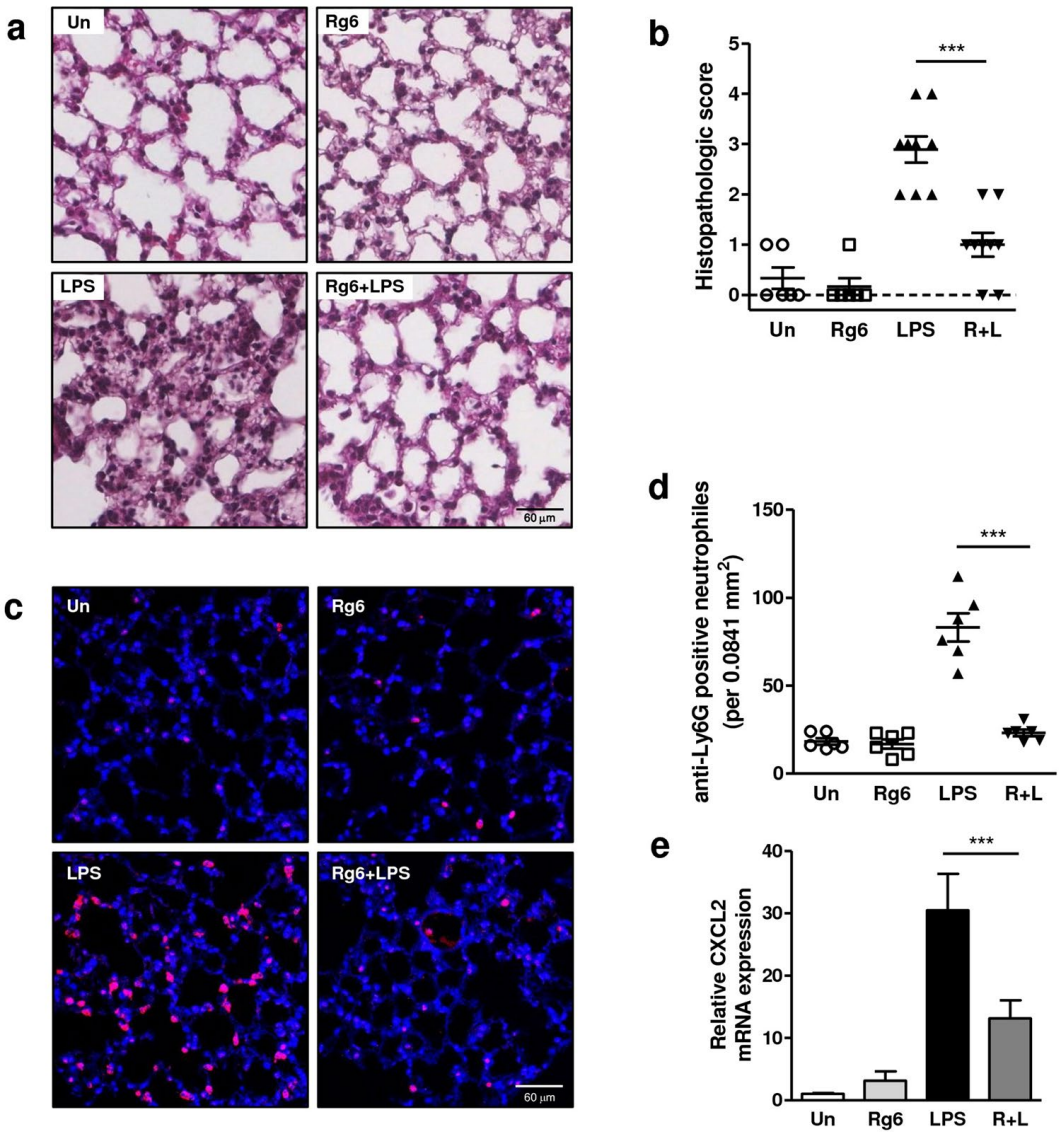
Ginsenoside Rg6 ameliorates lung damage in septic mice. (**a**) Mice were administered with LPS (30 mg/kg; IP) 2 h after Rg6 (20 mg/kg; IP) injection. The lungs from the mice were perfused, collected, and fixed at 6 h post-LPS treatment. The sectioned lung tissues were H&E-stained and the representative images from each group are shown (scale bar: 60 μm). (**b**) Histopathological scores were assigned as described in the Materials and Methods section. The severity of inflammation was graded by scanning multiple random fields in three sections of each lung tissue per mouse (n = 3 mice per group). (**c**) The lungs from mice were perfused, collected, and fixed at 6-h post-LPS injection. The sectioned lung tissues were placed onto slides, the neutrophils were immunostained with anti-Ly6G antibodies (purple), and cell nuclei were stained with DAPI (blue). Representative confocal images from each group are shown (scale bar: 60 μm). (**d**) The numbers of anti-Ly6G-positive neutrophils were manually counted from six random fields of confocal images in each group. **(e)** Relative CXCL2 chemokine expression was analyzed from the mRNA of lung tissues harvested from LPS-injected (30 mg/kg; IP) septic mice. The results are the means ± SD of at least six independent data points. Significant differences are indicated by asterisks (***P < 0.001). Un, Un-treated control; R + L, Rg6 and LPS addition.

To assess the Rg6 effect on the lung injury of the septic mice, a histopathological study was carried out 6 h after LPS injection. Unlike the phosphate-buffered saline (PBS)- or Rg6 only-injected mice, the lungs of LPS-injected mice displayed highly wounded characteristics, such as the infiltration of inflammatory cells, congestion, and alveolar wall thickening (Fig. 3a). Conversely, lungs from mice that had been pre-injected with Rg6 exhibited much less damage despite LPS injection. The lung pathologies of these four groups were each assigned a histopathological score (Fig. 3b). Additionally, we stained the remaining lung tissue using anti-Ly6G antibodies to determine whether there was difference in the number of neutrophils that infiltrated. As expected, significant changes were observed in the LPS-injected mice, depending on the presence of Rg6 (Fig. 3c). The number of anti-Ly6G antibody-stained neutrophils was counted and plotted (Fig. 3d). The tendency observed using Rg6 was again verified by measuring CXCL2 chemokine expression in mRNA extracted from lung cell lysates (Fig. 3e). Together, Rg6 improved the severity of lung damage and reduced excessive neutrophil infiltration in septic mice.

### Rg6 downregulates pro-inflammatory responses and upregulates IL-10 levels in mice with LPS-induced sepsis

**Figure 4:**
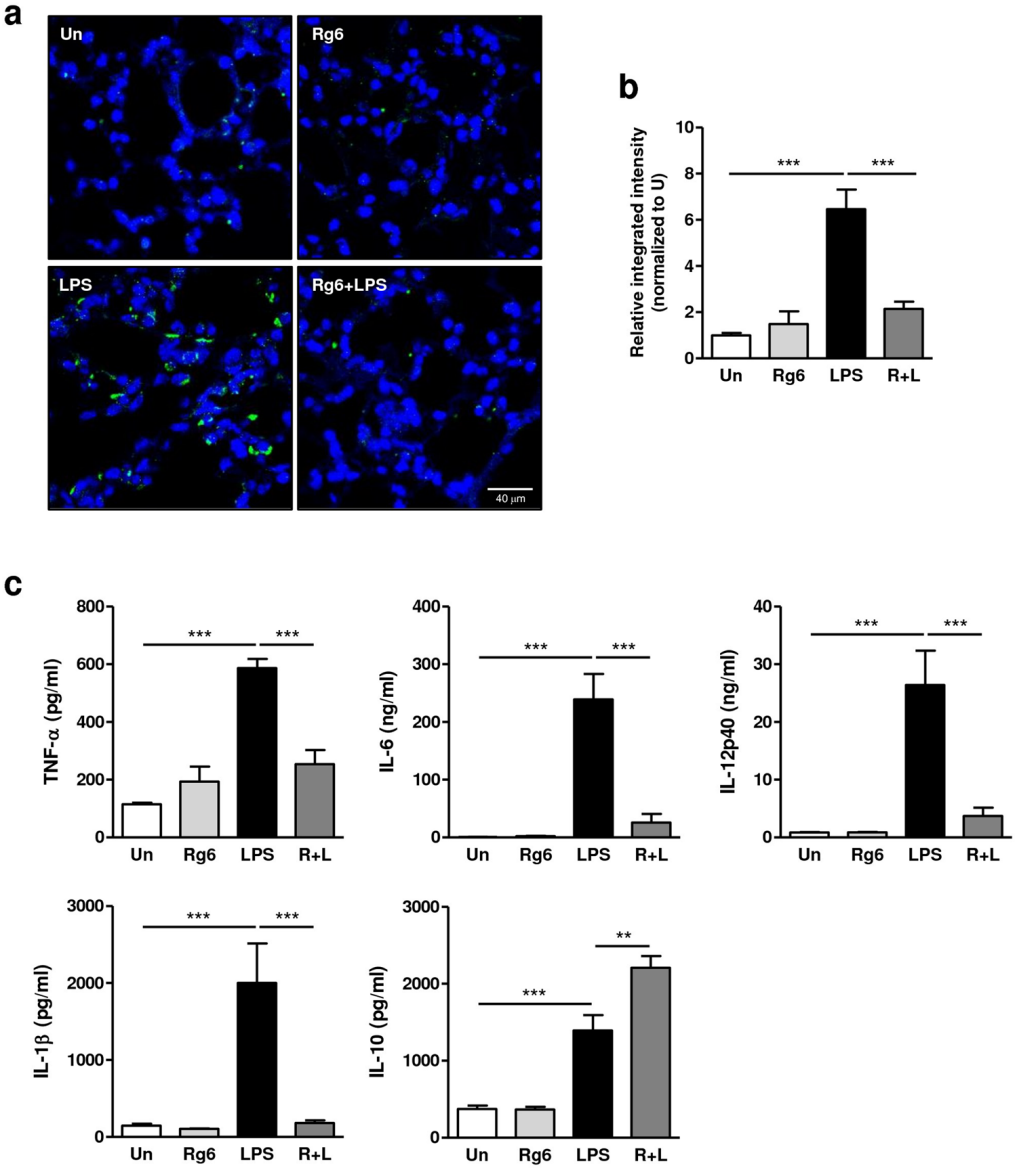
Ginsenoside Rg6 decreases pro-inflammatory responses and increases IL-10 secretion in septic mice. (**a**) Mice were administered with LPS (30 mg/kg; IP) 2 h after Rg6 (20 mg/kg; IP) injection. Mouse lungs were perfused, collected, and fixed at 18 h post-LPS treatment. The sectioned lung tissues were mounted onto slides and immunostained with anti-TNF-α antibodies (green) and DAPI (blue). Representative confocal images from each group are shown (scale bar: 40 μm). (**b**) The relative integrated intensity of TNF-α fluorescence from nine confocal images of each group was measured. (**c**) Mouse sera (n = 3 per group) were harvested at 18 h post-LPS injection (30 mg/kg; IP). Each serum was diluted appropriately, and the cytokine expression levels were analyzed using TNF-α, IL-6, IL-12p40, IL-1β, and IL-10 ELISA kits. The results are the means ± SD of at least six independent measurements. Significant differences are indicated by asterisks (**P < 0.01 and ***P < 0.001). Un, Un-treated control, R + L, Rg6 and LPS addition.

Because several ginsenosides are reported to have anti-inflammatory functions, we examined whether Rg6 could effectively inhibit LPS-induced systemic inflammation in mice. Rg6 and LPS were injected at the same doses reported in the previous procedure, and formalin-perfused lungs were collected 18 h after LPS injection. Paraffin blocks of lung tissue were sectioned and stained with anti- TNF-α antibodies to visualize by confocal microscopy. The numbers and intensities of TNF-α−stained puncta were significantly reduced in the group pre-injected with Rg6 compared to the group injected with only LPS (Fig. 4a). Their relative integrated intensities were measured and plotted (Fig. 4b).

To further investigate the effects of Rg6 on the LPS-induced cytokine storm, we retrieved serum from the mice by retro-orbital bleeding 18 h after LPS-injection. The collected serum was then diluted and subjected to enzyme-linked immunosorbent assay (ELISA) to measure the cytokine levels. The amounts of pro-inflammatory cytokines, such as TNF-α, IL-6, interleukin 12p40 (IL-12p40), and IL-1β, were significantly reduced in the Rg6-administered group, whereas the levels of the anti-inflammatory cytokine IL-10 increased (Fig. 4c). It was concluded that Rg6 effectively downregulated LPS-induced systemic inflammatory responses in the mouse model.

### Rg6 decreases LPS-induced pro-inflammatory responses in bone marrow-derived macrophages (BMDMs)

**Figure 5:**
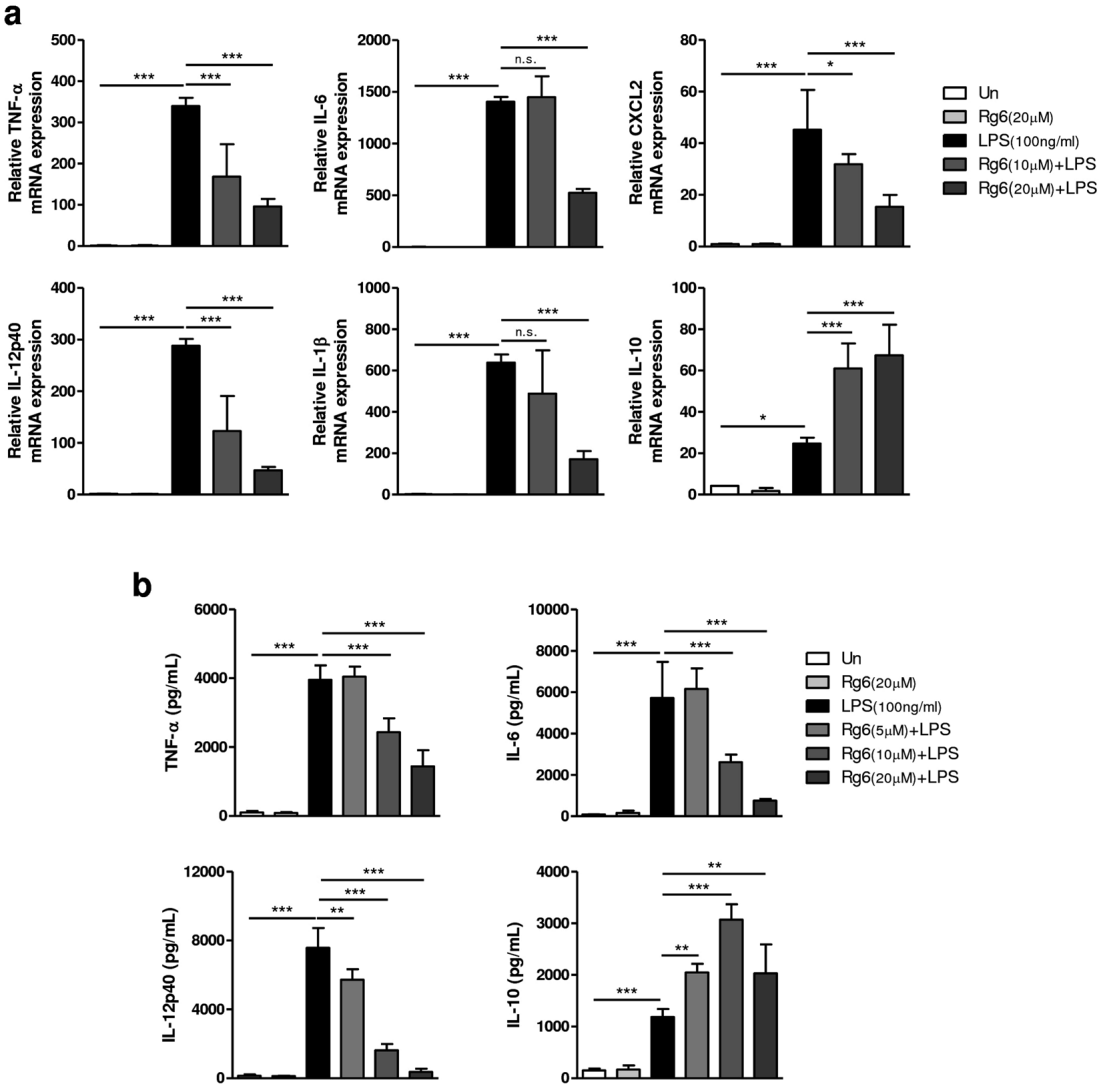
Ginsenoside Rg6 treatment decreases the expression of LPS-induced pro-inflammatory cytokines and increases the IL-10 level in BMDMs. (**a**) BMDMs were pre-treated with Rg6 (10 and 20 μM) for 1 h, followed by LPS treatment (100 ng/mL). After 6 h, the supernatants were removed and the cells were harvested to extract total RNA. The relative mRNA expression was analyzed using primers for TNF-α, IL-6, CXCL2, IL-12p40, IL-1β, and IL-10. (**b**) BMDMs were pre-treated with Rg6 (5, 10, and 20 μM) for 1 h, followed by LPS treatment (100 ng/mL). After 18 h, the supernatants were harvested and diluted appropriately. The protein expression levels were measured using TNF-α, IL-6, IL-12p40, and IL-10 mouse ELISA kits. The results are the means ± SD of at least four independent data points. Significant differences are indicated by asterisks (*P < 0.05, **P < 0.01, and ***P < 0.001). n.s., non-specific.

Macrophages play critical roles in the initiation, maintenance, and resolution of inflammation by activating signals such as cytokines^26^. To determine the anti-inflammatory effects of Rg6 under *in vitro* conditions, we used BMDMs as host immune cells. BMDMs were treated with LPS at 100 ng/mL to induce the inflammatory response, and the cells were pre-treated with Rg6 at various doses to assess whether the effects it conferred were dose-dependent. Cells were lysed and the extracted RNAs were subjected to quantitative real time PCR (qPCR) to determine the relative expression of cytokine mRNAs 6 h after LPS treatment (Fig. 5a). The expression of pro-inflammatory cytokine mRNAs, such as those for TNF-α, IL-6, IL-12p40, and IL-1β, was significantly decreased under Rg6-treated conditions in a dose-dependent manner compared to that in the LPS-treated group. The CXCL2 chemokine expression revealed the same tendency as that demonstrated with the pro-inflammatory cytokines, whereas IL-10 expression increased with the addition of Rg6.

Moreover, we identified the expression of mRNAs for TLR negative regulators^27^ and M2 macrophage markers^28^ (Supplementary Fig. S2). Mechanistically, Rg6 did not induce the TLR negative regulators A20 and IRAK-M in BMDMs. Instead, it slightly decreased the expression of LPS-induced A20, whereas there was no difference in the expression of IRAK-M. There was no apparent change in the expression of the M2 macrophage markers Mrc1 and Ym1 after Rg6 addition, indicating that the anti-inflammatory function of Rg6 was not induced by M2 polarization of macrophages. We also assessed the levels of secreted cytokines. Supernatants from the cultured BMDMs were collected and subjected to ELISA to measure cytokine levels 18 h after LPS treatment (Fig. 5b). It was again revealed that Rg6 addition could effectively decrease LPS-induced TNF-α, IL-6, and IL-12p40 secretion levels and increase LPS-induced IL-10 secretion levels. In summary, it was found that the addition of Rg6 decreased the LPS-induced inflammatory responses in macrophages, as revealed in both mRNA expression and protein secretion level. Rg6 also increased the levels of LPS-induced IL-10, as revealed by both gene expression and protein secretion, indicating that the increased levels of anti-inflammatory cytokine IL-10 likely inhibited the pro-inflammatory responses in the BMDMs.

### Rg6 decreases NF-κB activation and MAPK signaling pathways in BMDMs

**Figure 6:**
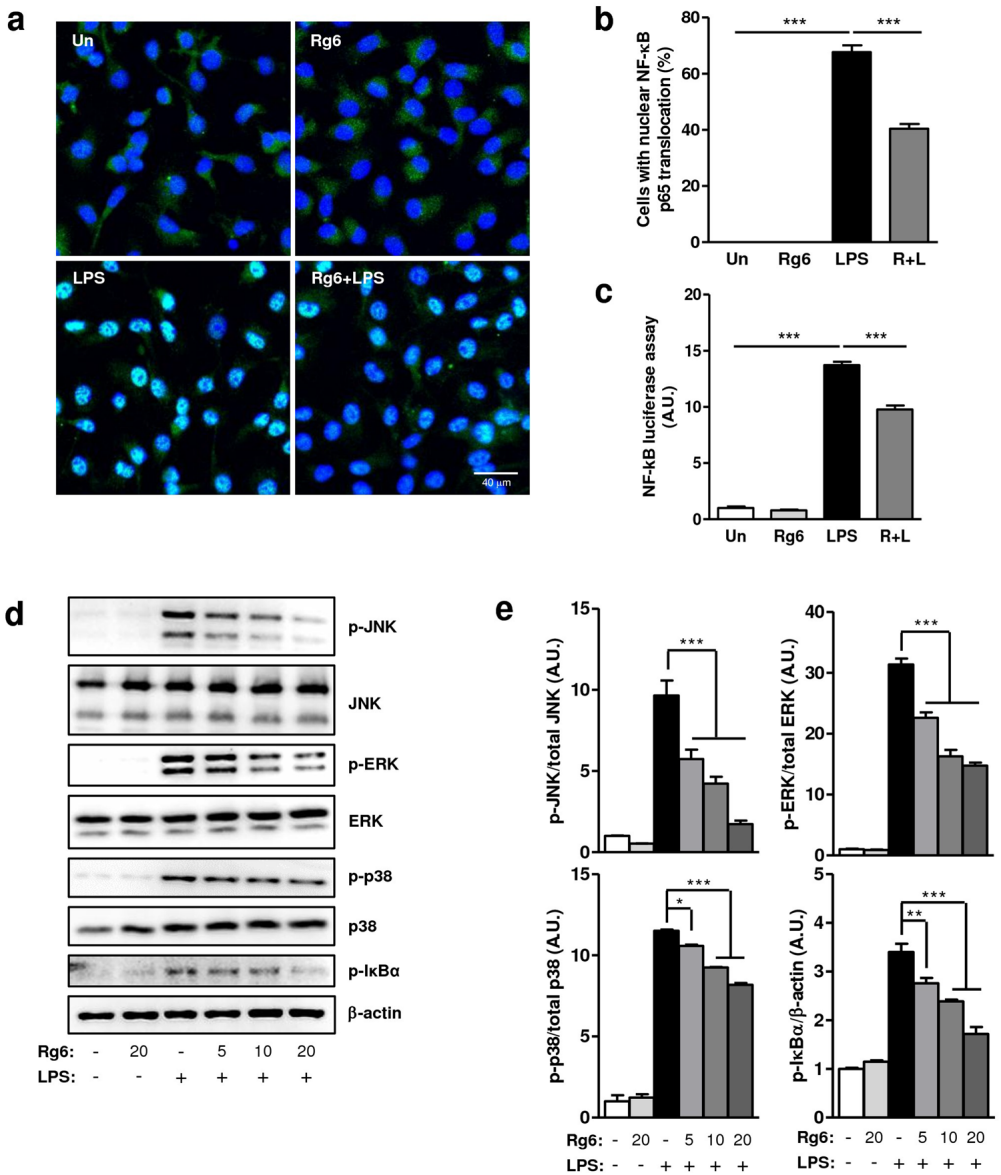
Ginsenoside Rg6 downregulates MAPK expression via inhibition of NF-kB signaling in BMDMs. (**a**) BMDMs were pre-treated with Rg6 (20 μM, 1 h), followed by LPS activation (100 ng/mL, 2 h). Nuclei were labeled with DAPI (blue) and p65 was immunostained using anti-p65 antibodies (green). The representative confocal images from each group were selected. (**b**) The percentage of cells with p65 translocated into the nucleus was manually calculated from confocal images shown in Fig. 4a. (**c**) BMDMs were transduced with NF-κB luciferase adenovirus for over 48 h. Thereafter, cells were pretreated with Rg6 (20 μM) for 1 h, and then LPS (100 ng/ml) was added for 6 h. The luciferase activity of the cell lysates was analyzed using the Luciferase Assay System. (**d**) BMDMs were pre-treated with Rg6 (5, 10, and 20 μM) for 1 h, followed by LPS (100 ng/mL) addition. After 30 min, the cells were lysed and subjected to immunoblotting using antibodies against p-JNK, JNK, p-ERK, ERK, p-p38, p38, p-IκBα and β-actin. The image is a representative of three experiments with similar results. To improve the clarity of the presentation, blot images were cropped. Full-length images of the same blots are presented in Supplementary Fig. S3. (**e**) Densitometric values of p-JNK, p-ERK, p-p38, and p-IκBα were normalized to those of JNK, ERK, p38, and β-actin, respectively. The results are the means ± SD of at least four independent data points. Significant differences are indicated by asterisks (*P < 0.05, **P < 0.01, and ***P < 0.001). Un, Un-treated control; R + L, Rg6 and LPS addition.

At the early stages of acute inflammation, it is well known that hindering NF-κB signaling results in the downregulation of mitogen-activated protein kinases (MAPKs) and inflammatory responses^29,30^. We assessed whether the anti-inflammatory effects of Rg6 affected the NF-κB signaling pathway. First, BMDMs were cultured on cover glass and stained with anti-p65 antibodies to determine the location of p65 within the cells using confocal microscopy. When the cells were treated with LPS, green-colored p65 was highly translocated into the 4′,6-diamidino-2-phenylindole (DAPI)-stained nuclei, displaying a bright sky-blue color. However, the amount of p65 translocated to the nuclei was diminished in the Rg6 and LPS-treated cells (Fig. 6a). The numbers of cells in which p65 was translocated into the nucleus were counted and the relative abundances of these cells were plotted. P65 nuclear translocation was observed in significantly fewer Rg6 LPS-treated cells than LPS-treated cells (Fig. 6b).

NF-κB signaling was also assessed using an adenovirus luciferase expression system. After transfecting adenovirus expressing p65 signal into BMDMs, the cells were treated with Rg6 and LPS and subjected to a luciferase assay system, and the intensity of luciferase was measured. The results further indicated that Rg6 decreased LPS-induced NF-κB signaling, as was observed in the p65 confocal imaging experiment (Fig. 6c). Next, we conducted western blot analysis to assess the effects of Rg6 on MAPK and IκBα activation. As the concentration of Rg6 used in the pretreatments increased, LPS-induced anti-phospho-JNK (p-JNK), anti-phospho-ERK1/2 (p-ERK), anti-phospho-p38 (p-p38), and anti-phospho-IκBα (p-IκBα) expression clearly decreased (Fig. 6d). The densities of p-JNK, p-ERK, p-p38, and p-IκBα were normalized to those of JNK, ERK, p38, and β-actin, respectively, revealing a significant reduction in each signal upon treatment with Rg6 (Fig. 6e). Therefore, the anti-inflammatory effects of ginsenoside Rg6 are partially due to its function in impeding the NF-κB signaling pathways.

### Rg6 induces the expression of miR-146a, an operator miRNA for anti-inflammation, in BMDMs

**Figure 7:**
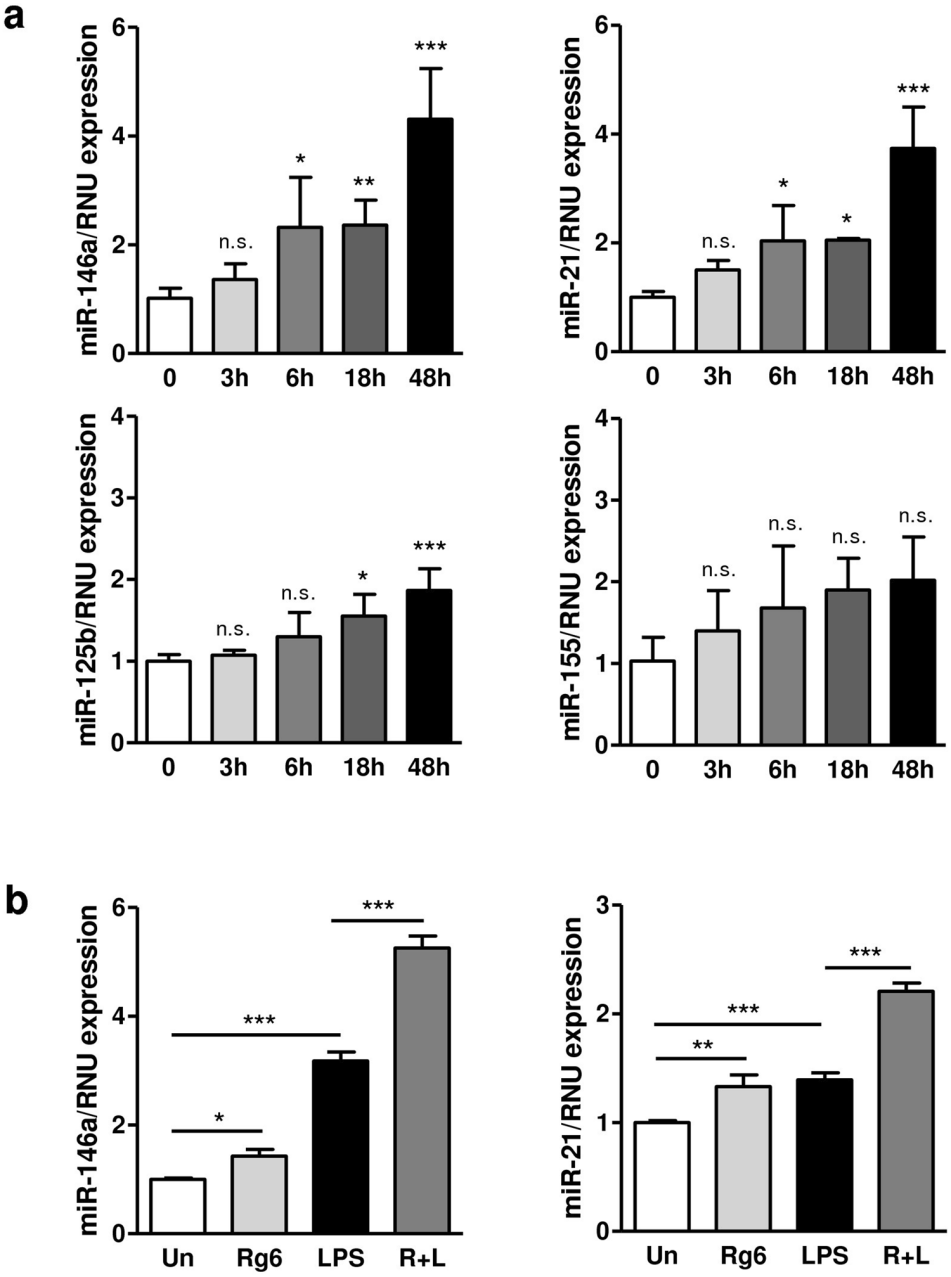
Ginsenoside Rg6 significantly induces miR-146a expression in BMDMs. (**a**) BMDMs were treated with Rg6 (20 μM), and time-dependent expression of miR-146a, miR-21, miR-125b, and miR-155 was analyzed. (**b**) Expression of miR-146a and miR-21 was evaluated in BMDMs treated with Rg6 (20 μM, 1 h prior to LPS addition) and LPS (100 ng/mL) for 18 h. The results are the means ± SD of at least four independent data points. Significant differences are indicated by asterisks (*P < 0.05, **P < 0.01, and ***P < 0.001). n.s., non-specific; Un, Un-treated control; R + L, Rg6 and LPS addition.

We demonstrated the anti-inflammatory functions of Rg6 and increased IL-10 secretion and inhibition of the NF-κB signaling pathway as its possible mechanisms. Furthermore, as miRNAs have recently emerged as a key post-transcriptional regulator of inflammation^14^, we hypothesized that Rg6 could directly induce anti-inflammatory miRNAs. We selected miR-146a, miR-21, miR-125b, and miR-155 as possible candidates and verified their expression at different time points following treatment with Rg6 in BMDMs (Fig. 7a). miR-146a expression was the most dominant in a time-dependent manner, while other miRNAs, such as miR-125b and miR-155, showed little or no specific increases at these experimental time points. Interestingly, the expression of miR-21 also showed a similar tendency with miR-146a.

Next, to assess miR-146a and miR-21 expression under inflammatory conditions, cells were treated with Rg6 and LPS and harvested at 18 h after LPS activation. The addition of Rg6 to cells in the LPS-induced inflammatory state significantly increased the expression of miR-146a and miR-21 to a greater extent than cells treated with LPS only (Fig. 7b). However, the fold change of miR-146a was greater than that of miR-21 under Rg6/LPS co-treated conditions compared to the unstimulated group. In short, miR-146a was the most critical miRNA regulating the Rg6-induced anti-inflammatory response in LPS-activated BMDMs among the tested miRNAs.

### Ginsenoside Rg6-mediated miR-146a expression is responsible for the inhibition of the LPS-induced production of pro-inflammatory cytokines

**Figure 8:**
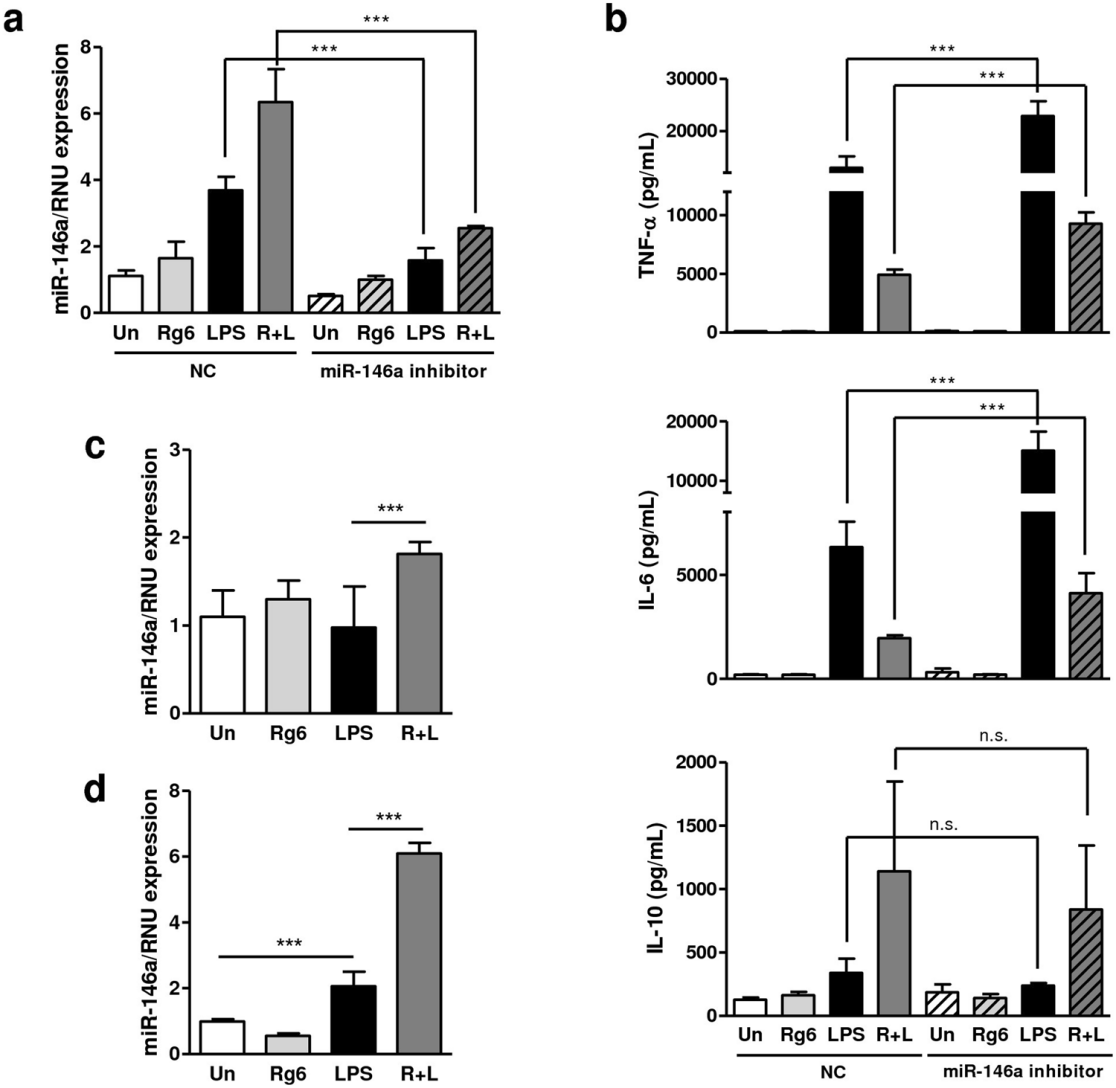
Ginsenoside Rg6-mediated miR-146a expression is responsible for the inhibition of the LPS-induced production of pro-inflammatory cytokines. (**a**) BMDMs were transfected with 50 nM of miR-146a inhibitor or its negative control (NC). After treating Rg6 (20 μM, 1 h prior to LPS addition) and LPS (100 ng/mL) for 18 h, miRNA was extracted from the cells to examine the miR-146a expression. (**b**) The supernatants from the transfected cells were harvested and subjected to ELISA to measure TNF-α, IL-6, and IL-10 production. (**c** and **d**) Mice were administered with LPS (30 mg/kg; IP) 2 h after Rg6 (20 mg/kg; IP) injection. Then, mouse lungs (**c**) and spleens (**d**) were collected and homogenized at 18 h post-LPS treatment. Homogenates were filtered, centrifuged, and the remaining cell pellets were subjected to miRNA extraction to examine the miR-146a expression. The results are the means ± SD of at least six independent data points. Significant differences are indicated by asterisks (***P < 0.001). n.s., non-specific; Un, Un-treated control; R + L, Rg6 and LPS addition; NC, negative control of miR-146a inhibitor.

It is known that miR-146a acts as a negative feedback regulator of LPS-induced inflammatory responses^18^. We thus aimed to investigate the function of miR-146a in the regulation of inflammatory responses in Rg6/LPS-treated BMDMs. We transfected a miR-146a inhibitor and its negative control (NC) into BMDMs and examined the miR-146a expression level. miR-146a expression was significantly downregulated in the inhibitor-transfected conditions when compared to NC (Fig. 8a). The secretion of pro-inflammatory cytokines TNF-α and IL-6 from the supernatants of the transfected macrophages was markedly increased by miR-146a inhibition (Fig. 8b; top and middle). However, the IL-10 level was not significantly regulated by miR-146a inhibition, suggesting that IL-10 is not a direct target of miR-146a under these conditions (Fig. 8b; bottom). In addition, miR-146a inhibition did not completely recover the production of TNF-α or IL-6 expression under Rg6/LPS-treated conditions compared to LPS-treated conditions (Fig. 8b; top and middle). These data suggest there are other mechanisms besides miR-146a by which Rg6 suppresses LPS-induced inflammation in macrophages. Together, our findings indicate that ginsenoside Rg6-mediated miR-146a expression is, at least in part, responsible for the inhibition of the LPS-induced production of pro-inflammatory cytokines, such as TNF-α and IL-6, in murine macrophages.

We also examined miR-146a expression levels in the lungs and spleens of septic mice treated with Rg6. Co-treatment of Rg6 and LPS led to a significant increase of miR-146a in the lungs (Fig. 8c) and spleens (Fig. 8d), when compared to that of the LPS-treated group. However, in the lungs, we did not observe an increase of miR-146a in samples from mice treated with LPS alone, presumably due to the difficulty associated with obtaining the optimal time points in the *in vivo* samples. Nevertheless, our experiments support Rg6-mediated induction of miR-146a in LPS-treated mice *in vivo*.

## Discussion

As the prevalence of immunocompromised patients increases due to impaired or weakened immune systems, the incidence of sepsis is growing worldwide^5^. However, management of sepsis by specific reversal of the cytokine storm or organ injury has been lacking until now. Steroids have been used against sepsis due to their various theoretical benefits, such as increased sensitivity of α and β adrenergic receptors, prevention of neutrophil aggregation, and decreased transcription of pro-inflammatory cytokines^31^. However, the clinical benefits of steroid treatment remain controversial. Although a recent clinical trial reported that mortality was lower in septic shock patients who received hydrocortisone plus fludrocortisone^32^, other studies have shown that steroid treatment did not improve the survival of patients in septic shock^33–35^. Thus, a new therapeutic approach for the treatment of sepsis is needed.

Ginseng components, which are known as ginsenosides, are promising candidates for the treatment of sepsis^36^. They are found exclusively in *Panax ginseng*^37^, a medical herb that has been used for over a thousand years in Far Eastern countries, with therapeutic uses that have been demonstrated in a number of medical conditions, such as cancer^38^, diabetes^39^, neurodegenerative disorders^40^, and inflammatory diseases^41^. Recent studies have focused on several effective ginsenosides, including Rb1^19,42^, Rh1^21^, Rg1^20,24^, Rg3^25^, and Rg5^43^. Indeed, a recent paper reported that ginsenoside Rg1 maintained the glucocorticoid efficacy in acute and chronic inflammatory conditions with reduced side effects, such as hyperglycemia or osteoporosis^20^. This finding demonstrates the potential of ginsenosides as novel anti-inflammatory agents that can overcome the limitations of steroids.

Ginseng can be classified as white ginseng (WG) or red ginseng (RG), according to the processing conditions^44^. WG is fresh ginseng which has been dried without being heated, while RG is steamed and dried ginseng which has reddish color. BG, manufactured by nine-times repeated steaming and drying of white ginseng has received attention from scientists because of its various pharmacological activities, such as anti-diabetic, wound healing, immune-stimulatory, and antioxidant properties^45–47^. Since ginsenoside, the main bioactive ingredient of ginseng, contains several sugars, such as glucose and rhamnose, the polarity and molecular weight (MW) of ginsenoside are very high, and the cell permeability of ginsenoside is very low. However, during the manufacturing process of BG, the structure of ginsenoside in white ginseng is transformed into low-molecular-weight and low-polarity rare ginsenosides by hydrolysis, isomerization, and dehydration at C-20, and hydrolysis also occurs at C-3 or C-6 in the aglycon skeleton^22^. Generally, the log octanol/water partition coefficient (log P) of a molecule reflects its ability to pass through the lipid bilayer; a molecule with a higher log P has more cell permeability^48^. According to the StarDrop program (version 6.5), the log P of ginsenoside Rg1 (MW: 801 Da) is 0.45, and the log P of ginsenoside Re (MW: 947 Da) is 0.24. Therefore, ginsenoside Rg1 can penetrate the cell membranes but ginsenoside Re cannot^49^. In the case of the rare ginsenoside Rg6 (MW: 767 Da), the log P value (1.29) is higher than that of ginsenoside Rg1 (0.45), suggesting that the rare ginsenoside Rg6 can easily enter inside cells.

The rare ginsenoside Rg6 is a specific PPT-type ginsenoside that exists only in BG, but not in WG or RG^22^. However, the amount of the rare ginsenoside Rg6 in BG is very low (0.19 mg in 1 g of BG). In addition, as there are over 30 types of ginsenosides in BG, the isolation and purification of the single ginsenoside Rg6 from BG extracts is difficult and time-consuming. Therefore, it is important to establish a mass production process for the desired ginsenoside as well as to find new effective material. We recently developed a technique for the mass production of the rare ginsenoside Rg6 from the ginsenoside Re, which is a main component of ginseng leaves^50^. Using this method, Rg6 was sufficiently prepared, and our group reported its effects on tumorigenesis^51^.

Although *in vivo* studies with very rare ginsenosides are limited, we were able to test the effects of Rg6 on overall survival of mice with LPS- and CLP-induced septic shock. As shown in Fig. 2, the *in vivo* survival rate changed more dramatically in the LPS-induced sepsis model than in the CLP-induced model when Rg6 was pre-injected. This difference may be due to the different components involved in the activation of inflammation in each model. In the LPS-induced sepsis model, only the LPS stimulates the inflammatory response in the host, whereas CLP-induced sepsis is stimulated by various microorganisms. Thus, the reduction in TNF-α may have affected the anti-bacterial effects in the CLP-induced model, resulting in weakened host protection against bacterial infection^52,53^. Another possible explanation is that the activity of Rg6 primarily inhibits the TLR4 pathway rather than the pathways of other TLRs, which is supported by reports that ginsenosides Re and Rg5 directly inhibited the binding of LPS to TLR4 on macrophages^23,43^. However, our findings reveal that Rg6 can effectively counteract both LPS- and CLP-induced sepsis and ameliorate the survival rate in mice. In addition, as there was a significant difference in survival between the LPS control and Rg6 post-treated groups, our data strongly suggest that Rg6 has therapeutic potential over endotoxemia and would be a good candidate for the treatment of sepsis. Although we confirmed that Rg6 exhibited significant activity via IP injection in the amelioration of systemic inflammation, oral administration of Rg6 did not improve mouse survival relative to the vehicle control (data not shown). Further studies are warranted to develop an oral drug delivery system that would be the most convenient, popular, and cost-effective route of drug administration.

Our data showed that Rg6 had a significant inhibitory effect upon LPS-induced inflammatory responses and signaling including NF-κB and MAPK pathways in BMDMs. Similarly, Rg6 treatment led to a reduction of LPS-induced inflammatory cytokine generation in peripheral blood leukocytes from mice and humans (data not shown). To examine the mechanisms of Rg6-mediated anti-inflammatory effects, we focused on miRNAs, which are small non-coding RNA molecules that function in RNA silencing and post-transcriptional regulation of gene expression^54^. Among the miRNAs, mature miR-146a is reported to directly target the IRAK1 and TRAF6 molecules and to affect the translation of pro-inflammatory cytokine mRNAs^14,55^. We have revealed that treatment with Rg6 significantly increased miR-146a levels in macrophages. When macrophages are treated with LPS, it is thought that miR-146a is expressed to counteract the overexpression of pro-inflammatory cytokines to maintain homeostasis^18,56^. In response to the treatment of Rg6 to the LPS-activated cells, miR-146a expression was additively increased and the levels of inflammatory cytokines such as TNF-α and IL-6 were significantly reduced. The mechanism by which ginsenoside Rg6 regulates both miR-146a and IL-10 expression has yet to be elucidated. It is known that NF-κB activity is required for miR-146a elevation in macrophages^14,18^. In previous studies of endotoxin tolerance, the activation of miR-146a can be regulated by C/EBPβ, which is critically involved in the mechanism of endotoxin tolerance^57^. In addition, numerous studies have shown that IL-10 production is mediated through the activation of C/EBPβ^58–60^. Future studies are needed to examine whether Rg6-mediated increase of C/EBPβ might contribute to the biogenesis of miR-146a as well as IL-10 in LPS-treated BMDMs.

We found that at the highest concentration of Rg6 (20 µM), there was some inconsistency of Rg6 regulation in IL-10 mRNA and protein expression (see Fig. 5a and b). These data strongly indicate that Rg6 may regulate IL-10 production at the post-transcriptional level. miR-146a did not directly regulate IL-10 production in BMDMs co-treated with Rg6/LPS. Previous studies have reported that the transfection of pro-miR-21 resulted in the production of IL-10, which was induced by LPS^61^. Combined with our data that Rg6 induces miR-21 as well as miR-146a (see Fig. 7a and b), these data suggest that another miRNA, such as miR-21, may play a role in the post-transcriptional regulation of IL-10 in BMDMs.

In summary, this study has revealed the potential uses of ginsenoside Rg6 as a new anti-inflammatory agent to counteract the inflammatory state in septic mice. Our data strongly suggest that the advantage of Rg6 in the treatment of sepsis is the reversal of lung damage and the reduction in excessive inflammatory cytokines. Moreover, the unique function of Rg6 in the upregulation of miR-146a in macrophages offers the possibility of a new therapeutic approach for the treatment of sepsis. Further studies in clinical settings will provide more insight into the functional roles of ginsenoside Rg6 in the treatment of inflammatory diseases.

## Materials and Methods

### Preparation of rare ginsenoside Rg6

Pure ginsenoside Rg6 was easily prepared from single ginsenoside Re, largely isolated from ginseng leaf, instead of BG extracts according to previously reported method^50^. Briefly, the purified ginsenoside Re (10 g) in water (15 mL) was steamed at 120 °C for 6 h in an autoclave. The steamed ginsenoside Re was chromatographed on a RP-C 18 by MPLC (column Biotage SNAP Cartridges, Ultra C 18, 120 g X 2) eluting with Acetonitrile-Water (10–45% Acetonitrile, 10 L) to obtain pure rare ginsenoside Rg6 (2.28 g) with >98% purity. The purity of ginsenoside was analyzed by HPLC system (Supplementary Fig. S1). HPLC analysis was carried out on an Agilent Technologies 1260 infinity UV visible spectrometer and an ACE 5-C18 column (250 × 4.6 mm) was used at 40 °C. The binary gradient elution system consisted of water (A) and acetonitrile (B). The separation was achieved using the following gradient program; water (A), acetonitrile (B); 0~3 min (20% B), 3~15 min (23% B), 15~20 min (33% B), 20~45 min (40% B), 45~60 min (68% B), 60~70 min (85% B), 70~75 min (20% B). The solvent flow rate was held constant at 1 mL/min and sample injection volume was 10 μL.

### Animals

C57BL/6 mice that were 6 to 8 weeks old with wild type background were purchased from Samtako Bio Korea (Gyeonggi-do, Korea), and were maintained under barrier conditions in a biohazard animal room at the Medical Research Center of Chungnam National University, Daejeon, Korea. The animals were fed a sterile commercial mouse diet and were provided water *ad libitum*. All animal experiments were approved by the Institutional Research and Ethics Committee at Chungnam National University School of Medicine (CNU-00944; Daejeon, Korea). All animal-related procedures were performed in accordance with the guidelines of the Korean Food and Drug Administration.

### Mouse sepsis models

The mice used for the sepsis models were 8–10-week-old females. The CLP sepsis model was established following a previously reported method^62^ with slight modification. Briefly, mice were anesthetized using avertin, which was formulated by dissolving 0.5 g 2,2,2-tribromoethanol (Sigma-Aldrich, T48402) in 1 mL of 2-methyl-2-butanol (Sigma-Aldrich, 240486). PBS was added to prepare a working solution of 1.25% avertin, which was filtered using a 0.22-μm syringe filter (Merck, SLGP033RS) prior to use. After disinfecting the abdomen with 70% ethanol, a small midline incision was made, and the cecum was exposed. The cecum was ligated below the ileocecal valve (about 75% of total cecum length) with Black Silk (Ailee, SK447), punctured twice with an 18-gauge needle, and the abdomen was closed. The wound was sutured with 5-0 Surgifit (Ailee, AV521). The sham control mice were operated on without performing the ligation and puncture process. The LPS sepsis model was established by injecting 30 mg/kg of PBS-diluted LPS (Sigma-Aldrich, L3755) intraperitoneally. Rg6 (20 mg/kg) was dissolved in PBS and IP injected into each mouse once (2 h prior to LPS) for the pre-treat, and twice (1 and 2 h post-LPS injection) for the post-treat system.

### Histology

Lungs were harvested from the mice and fixed with 10% formalin overnight (O/N), and then embedded in paraffin wax. Paraffin blocks were then cut into 4-mm slices and stained with hematoxylin and eosin (H&E). The stained slides were scanned and imaged using a ScanScope CS System (Aperio Technologies). The severity of inflammation was graded by scanning multiple random fields in three sections of each lung tissue per mouse. An overall histopathological score was assigned to each section based on the extent of granulomatous inflammation as follows: 0 = no lesion, 1 = minimal lesion (<10% of area involved), 2 = mild lesion (10–30% area involved), 3 = moderate lesion (30–50% area involved), 4 = marked lesion (50–80% area involved), and 5 = severe lesion (>80% area involved), as described previously^63^.

### Immunohistochemistry

The paraffin blocks of fixed lung tissue were sectioned (4 mm) onto microscope slides and incubated at 60 °C for 30 min. The slides were then immersed in xylene twice, and in 100% and 70% ethanol. After removing all remaining paraffin, the slides were rinsed with tap water and distilled water. To enhance the staining intensity, the washed slides were heated in pH 6 citrate buffer at 118 °C for 20 min. The slides were blocked with peroxidase blocking buffer for 10 min, and then immunostained with antibodies specific for anti-mouse Ly6G (Bio X Cell, BP0075-1) and TNF-α (Santa Cruz, sc-52746) O/N. After washing, Alexa Fluor 488-conjugated anti-mouse IgG (Life Technologies, A-11029) and Alexa Fluor 594-conjugated anti-rat IgG (Life Technologies, A-11007) were used as secondary antibodies and bound for 2 h. To suppress auto-fluorescence, Sudan Black B treatment was performed, and the slides were mounted with DAPI (Sigma-Aldrich, D9542) for nuclear counterstaining. Confocal images of neutrophils and TNF-α visualization were taken with a Leica TCS SP8 confocal system and processed with Leica Application Suite X software. The number of anti-Ly6G-positive neutrophils was counted manually from six random fields of confocal images and the integrated intensity of TNF-α fluorescence from multiple confocal images of each group was measured using Metamorph NX 2.0 software.

### ELISA

The mice were anesthetized and blood was collected by retro-orbital bleeding at 18 h after injection with LPS. Collected blood was held in a vacutainer (BD, 367955) and incubated for 30 min at room temperature (RT). The samples were centrifuged at 3000 rpm for 5 min and the serum was retrieved as the supernatant. The serum was diluted in ELISA assay diluent at the appropriate proportion and analyzed using a Mouse BD OptEIA Set ELISA Kit (BD Biosciences) to detect TNF-α (558534), IL-6 (555240), IL-12p40 (555165), IL-1β (559603), and IL-10 (555252). For *in vitro* sample collection, supernatants of cultured BMDMs were harvested 18 h after LPS treatment, and diluted appropriately for each cytokine assay. All assays were performed following the manufacturers’ instructions. The commercial ginsenosides including Rg6 (CFN90565), Rg1 (CFN99967), Rg3 (CFN99969), and Re (CFN99974) were purchased from ChemFaces.

### Cell culture

Mouse bone marrow cells were isolated from the femurs and tibias of C57BL/6 mice (5–6 weeks old) and differentiated to BMDMs by culturing in DMEM (Serana, MCL-002) supplemented with 10% FBS (Serana, S-FBS-US-015) and 25 μg/mL M-CSF (R&D Systems, 416-ML) for 4–5 d at 37 °C and a 5% CO_2_ atmosphere.

### RNA extraction and qPCR

For *in vivo* samples, lung tissue suspended in PBS was homogenized and centrifuged to retrieve cell pellets before maintenance at −80 °C. *In vitro* samples were harvested by removing the supernatants from cultured BMDMs. Total RNA was extracted using TRIzol reagent (Thermo Fisher Scientific, 15596026) and used for synthesis of complementary DNA (cDNA) with Reverse Transcriptase Premix (Elpisbio, EBT-1515). Next, qPCR was carried out using cDNA, primers, and SYBR Green Master Mix (Qiagen, 204074) following the manufacturer’s protocol. Reactions were conducted on a Rotor-Gene Q 2plex system (Qiagen) and qPCR data were analyzed using the delta-delta CT relative quantification method with the Rotor-Gene 6000 Series software. Data were expressed as relative fold changes compared to the expression of the control gene *β-*actin. The primers used are summarized in Supplementary Table S1.

### NF-κB p65 translocation

Translocation of NF-κB into the nucleus was detected using immunofluorescence staining as described previously^64^. Briefly, BMDMs were treated with Rg6 (20 μM, 1 h prior to LPS addition) and LPS (100 ng/mL) for 2 h, followed by fixation in 4% paraformaldehyde in PBS for 10 min. Cells were then permeabilized with 0.25% Triton X-100 in PBS for 10 min and blocked with 10% BSA in PBS for 1 h. Rabbit NF-κB p65 antibody (Santa Cruz Biotechnology, sc-372) was diluted 1:400 and added to the cells at 4 °C O/N. After washing off the non-bound antibodies, cells were stained with anti-rabbit IgG Alexa Fluor 488 (Invitrogen, A-11008) for 1 h at RT. BMDMs on cover glass were mounted with DAPI to counterstain the nucleus and confocal images were taken using a Leica TCS SP8 confocal system.

### NF-κB luciferase reporter assays

NF-κB luciferase reporter assays were performed as previously reported^63^. Briefly, a NF-κB luciferase adenovirus (Viral Vector Core Facility) was transduced into BMDMs for 24 h, and maintained in 5% FBS containing DMEM for another 24 h. Pre-treatment with Rg6 (20 μM) was performed for 1 h and then treatment with LPS (100 ng/mL) was performed for 6 h. The cells were thoroughly washed with PBS at RT and cell lysates were prepared by dissolving cells in Cell Culture Lysis Reagent (Promega, E1531). Luciferase activity was analyzed using the Luciferase Assay System (Promega, E1501), according to the manufacturer’s instructions.

### Immunoblotting

BMDMs were harvested and lysed in RIPA buffer (LPS solution, CBR002) containing Protease Inhibitor Cocktail (Roche, 11697498001) on ice, followed by centrifugation at 15,000 rpm for 10 min at 4 °C to remove insoluble pellets. The prepared samples were subjected to SDS-PAGE, transferred to polyvinylidene difluoride membranes (Millipore, IPVH00010), blocked with 5% skim milk Tris-buffered saline Tween 20 buffer, and incubated O/N with the appropriate antibodies. Anti-β-actin (4970), anti-p-ERK (9101), anti-ERK (9102), anti-p-p38 (9211), anti-p38 (9212), anti-p-JNK (4671), anti-JNK (9252), and anti-p-IκBα (2859) primary antibodies were purchased from Cell Signaling Technology. After washing and incubating for 2 h with anti-rabbit HRP-linked secondary antibody (Cell Signaling Technology, 7074), enhanced chemiluminescence substrate (Millipore, WBKLS0500) was used to visualize proteins and was detected using a UVitec Alliance mini-chemiluminescence device (UVitec, Rugby, UK). The densitometric values were calculated using ImageJ software.

### Extraction and purification of microRNA (miRNA)

The extraction and purification of miRNA was carried out using the miRNeasy Mini Kit (Qiagen, 217004). To quantify mature miRNA expression, cDNA was synthesized from purified miRNA using the miScript II RT Kit (Qiagen, 218161). qPCR was performed using the miScript SYBR Green PCR Kit (Qiagen, 218073) with an initial activation step of 95 °C for 15 min, followed by cycling conditions of 50–60 cycles of 94 °C for 15 s, 55 °C for 30 s, and 70 °C for 30 s. The primers for miR-146a (Qiagen, MS00001638), miR-21 (Qiagen, MS00011487), miR-125b (Qiagen, MS00005992), miR-155 (Qiagen, MS00001701), and RNU6-2 (Qiagen, MS00033740) were purchased from Qiagen, and RNU6-2 was used for normalization. The inhibitor of miR-146a and its negative control (NC) were ordered from and manufactured by Genolution (Seoul, Korea). The inhibitor and NC were transfected into BMDMs at a final concentration of 50 nM using Lipofectamine 2000 Reagent (Invitrogen, 11668019) following the manufacturer’s protocol. To examine the miRNA expression levels *in vivo*, the lungs and spleens of C57BL/6 mice were harvested at 18 h post LPS administration and homogenized in 1 ml of PBS using Omni homogenizing kit (OMNI international, TH220-PCRD). Homogenates were filtered through a cell strainer (Falcon, 352350), and 100 μl of filtered samples were centrifuged at 3,000 rpm for 5 min. Residual cell pellets were then subjected to miRNA extraction process as described above.

### Statistical analysis

All experiments were repeated at least three times, with consistent findings. The significance of the differences between the survival curves was determined by the log-rank (Mantel-Cox) test, and that among three or more groups was analyzed by one-way analysis of variance followed by Bonferroni’s multiple comparison test using GraphPad Prism 5 software. Data are expressed as means ± standard deviation (SD); *P < 0.05, **P < 0.01, and ***P < 0.001 were considered indicative of statistical significance.

## Supplementary information

Supplementary Information

## Data Availability

All data generated or analysed during this study are included in this published article (and its Supplementary Information files).
