# Supplementary material for: Rg6, a rare ginsenoside, inhibits systemic inflammation through the induction of interleukin-10 and microRNA-146a
Source: Sci Rep. 2019 Mar 13;9:4342. doi: 10.1038/s41598-019-40690-8 (PMC6416268; doi:10.1038/s41598-019-40690-8)
Supplement: Supplementary file 1 — Supplementary Information [file 41598_2019_40690_MOESM1_ESM.pdf]

SUPPLEMENTARY INFORMATION

Rg6, a rare ginsenoside, inhibits systemic inflammation through the induction of interleukin-10 and microRNA-146a

Seungwha Paik[1,2,3], Jin Ho Choe[1,2,3], Ga-Eun Choi[3,4], Ji-Eun Kim[3,4], Jin-Man Kim[2,3,5], Gyu Yong Song[3,4*] & Eun-Kyeong Jo[1,2,3*]

[1]Department of Microbiology, Chungnam National University School of Medicine, Daejeon 35015, Republic of Korea; [2] Department of Medical Science, Chungnam National University School of Medicine, Daejeon 35015, Republic of Korea; [3]Infection Control Convergence Research Center, Chungnam National University, Daejeon 35015, Republic of Korea; [4]College of Pharmacy, Chungnam National University, Daejeon 34134, Republic of Korea; [5]Department of Pathology, Chungnam National University School of Medicine, Daejeon 35015, Republic of Korea;

Seungwha Paik, Jin Ho Choe & Ga-Eun Choi: These authors contributed equally to this work.

To whom correspondence should be addressed: Gyu Yong Song and Eun-Kyeong Jo
College of Pharmacy, Chungnam National University, Daejeon 34134, Republic of Korea . Phone: 82-42-821-5926. Fax: 82-42-823-6566. E-mail: gysong@cnu.ac.kr
Department of Microbiology, Chungnam National University School of Medicine, Daejeon 35015, Republic of Korea. Phone: 82-42-580-8243. Fax: 82-42-585-3686. E-mail: hayoungj@cnu.ac.kr

Supplementary Figure S1

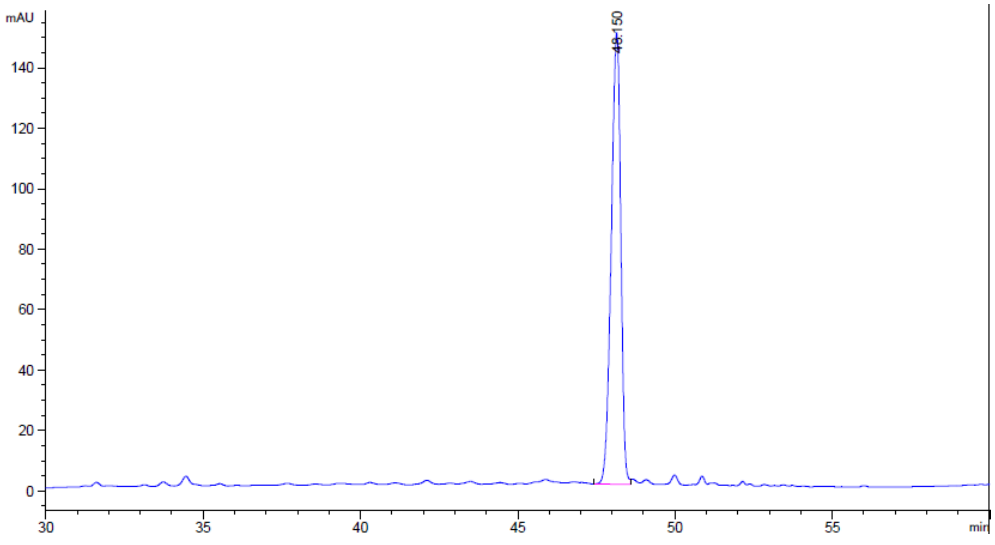

Supplementary Fig. S1. HPLC chromatograms of ginsenoside Rg6 (purity; 98%). The manufactured ginsenoside Rg6 was analyzed using HPLC system fitted with a C-18 column utilizing a solvent gradient system.

Supplementary Figure S2

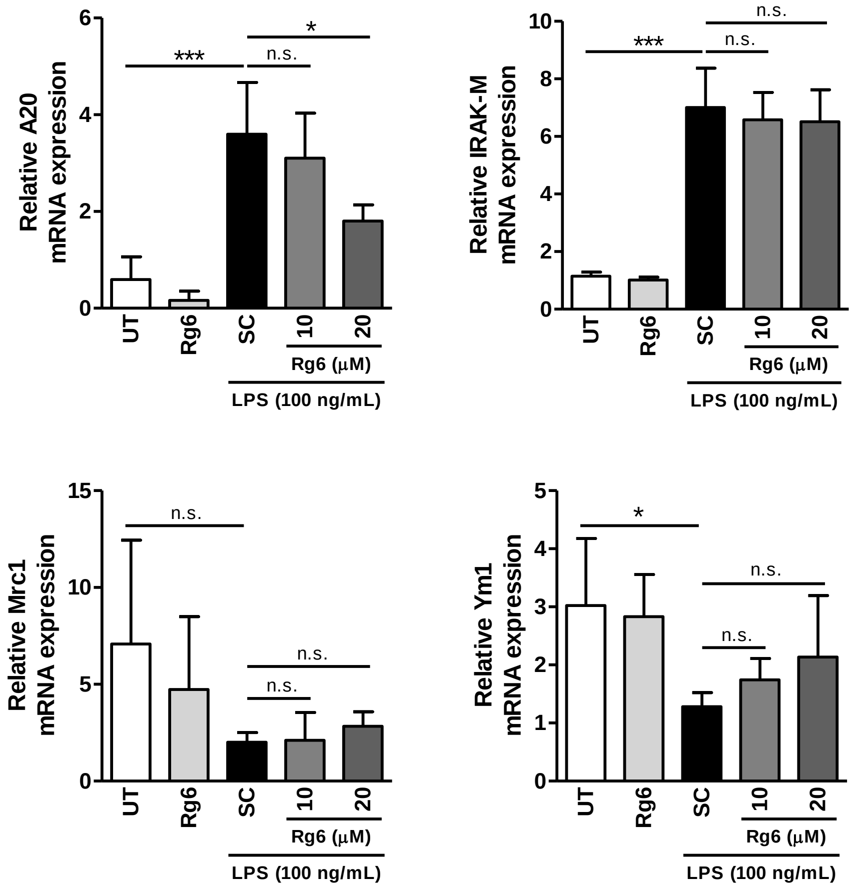

Supplementary Fig. S2. Rg6 did not induce the TLR negative regulators, such as *A20* and *IRAK-M*, nor M2 macrophage markers, such as *Mrc1* and *Ym1*. BMDMS were pre-treated with Rg6 (10 or 20 μM) for 1 h, followed by LPS treatment (100 ng/mL). After 6 h, the supernatants were removed and the cells were harvested to extract total RNA. The relative mRNA expression was analyzed using primers for *A20, IRAK-M, Mrc1,* and *Ym1*.

Supplementary Figure S3

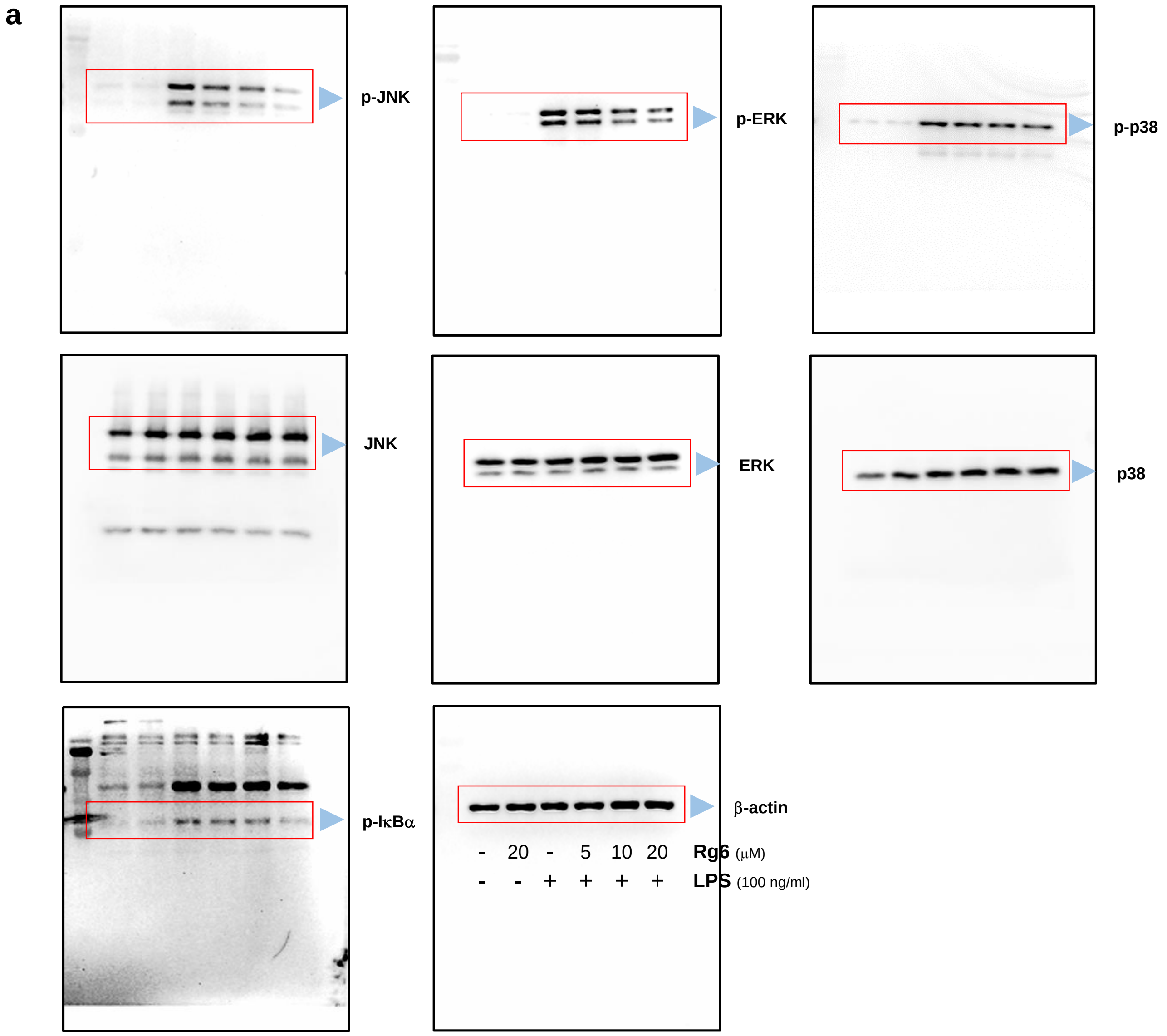

Supplementary Fig. S3. Full-length western blots of MAPKs and IκBα expression.
(a) Full-length images of the blots presented in the Fig. 6d. (b) Full-length blots were merged with membrane images to visualize size marker.

Supplementary Figure S3 (continued)

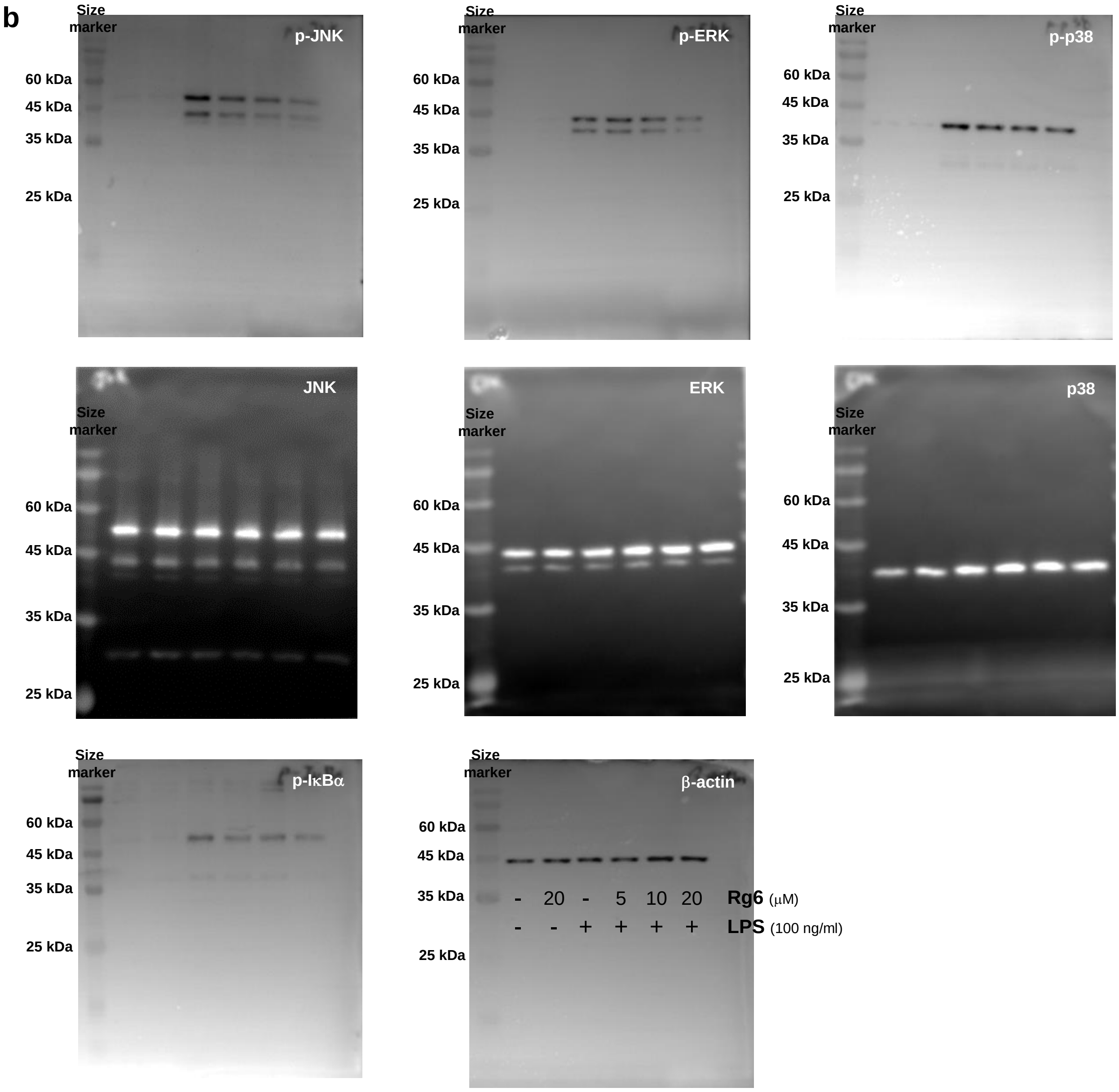

Supplementary Fig. S3. Full-length western blots of MAPKs and IκBα expression.
(a) Full-length images of the blots presented in the Fig. 6d. (b) Full-length blots were merged with membrane images to visualize size marker.

Supplementary Table S1

Gene name	Forward primers (5′-3′)	Reverse primers (5′-3′)
TNF-α	AGC ACA GAA AGC ATG ATC CG	CTG ATG AGA GGG AGG CCA TT
IL-6	ACA AAG CCA GAG TCC TTC AGA	TGG TCC TTA GCC ACT CCT TC
IL-12p40	AGG TCA CAC TGG ACC AAA GG	TGG TTT GAT GAT GTC CCT GA
IL-1β	TGA CGG ACC CCA AAA GAT GA	AAA GAC ACA GGT AGC TGC CA
IL-10	GCT CTT GCA CTA CCA AAG CC	CTG CTG ATC CTC ATG CCA GT
CXCL2	CAT CCA GAG CTT GAG TGT GAC G	GGC TTC AGG GTC AAG GCA AAC T
β-actin	CCA CCA TGT ACC CAG GCA TT	AGG GTG TAA AAC GCA GCT CA
A20	AAA CCA ATG GTG ATG GAA ACT G	GTT GTC CCA TTC GTC ATT CC
IRAK-M	AGC CAG TCT GAG GTC ACC TTT C	CGT TGC AAT CCG CTT CAC T
Mrc1	CTC TGT TCA GCT ATT GGA CGC	CGG AAT TTC TGG GAT TCA GCT TC
Ym1	AGA AGG GAG TTT CAA ACC TGG T	GTC TTG CTC ATG TGT GTA AGT GA

Supplementary Table. S1. Mouse primers used for qPCR analysis.
